# SNHG18 inhibits bladder cancer cell proliferation by increasing p21 transcription through destabilizing c-Myc protein

**DOI:** 10.1186/s12935-023-02887-w

**Published:** 2023-03-16

**Authors:** Meixia Ke, Ning Sun, Zhenni Lin, Peipei Zhang, Yan Hu, Shuilian Wu, Zhijian Zheng, Yongyong Lu, Honglei Jin

**Affiliations:** 1 Zhejiang Provincial Key Laboratory of Medical Genetics, Key Laboratory of Laboratory Medicine, Ministry of Education, School of Laboratory Medicine and Life Sciences, Wenzhou Medical University, Wenzhou, 325035 Zhejiang China; 2 Clinical Laboratory, Dongyang People’s Hospital, Dongyang, 322100 Zhejiang China; 3 Department of Urology, The First Affiliated Hospital of Wenzhou Medical University, Wenzhou, 325035 Zhejiang China

**Keywords:** lncRNA, SNHG18, c-Myc, p21, Bladder cancer

## Abstract

**Background:**

Long non-coding RNAs (lncRNAs) have been confirmed to play important roles in various cancers including bladder cancer (BC). The precise expression pattern of lncRNA small nucleolar RNA host gene 18 (SNHG18) in BC and its mechanisms of action have not been fully explored.

**Materials and methods:**

The expression of SNHG18 was evaluated by RT-qPCR in bladder cancer clinical samples and human bladder cancer cell lines, and stable cell lines overexpressing SNHG18 were constructed. The effect of SNHG18 on the proliferation of bladder cancer cells was detected by soft agar colony formation test, ATP activity test and subcutaneous tumorigenesis model in nude mice. The specific mechanism of SNHG18 inhibition of bladder cancer proliferation was studied by flow cytometry, western blotting, dual luciferase reporter gene assay and protein degradation assay.

**Results:**

We found that SNHG18 is significantly downregulated in BC tissues and cell lines. Kaplan–Meier analysis showed that SNHG18 expression is positively correlated with survival in BC patients. Ectopic overexpression of SNHG18 significantly inhibited the proliferation of BC cells in vitro and in vivo. Further mechanistic investigations demonstrated that SNHG18 inhibited c-Myc expression by modulating the ubiquitination-proteasome pathway and that c-Myc is the critical transcription factor that mediates SNHG18 inhibition of BC growth by directly binding to the p21 promoter, which was attributed with significant p21 accumulation.

**Conclusions:**

SNHG18 promotes the transcription and expression of p21 by inhibiting c-Myc expression, leading to G0-G1 arrest and inhibiting the proliferation of bladder cancer cells. These findings highlight a novel cell cycle regulatory mechanism involving the SNHG18/c-Myc/p21 pathway in BC pathogenesis and could potentially lead to new lncRNA-based diagnostics and/or therapeutics for BC.

**Supplementary Information:**

The online version contains supplementary material available at 10.1186/s12935-023-02887-w.

## Introduction

Bladder cancer (BC) is one of the most commonly diagnosed cancers of the genitourinary system [1]. The latest data show that there were 573,278 new cases of BC in 2020, making it the fourth most common tumor worldwide [2]. When BC is diagnosed in advanced stages or with metastasis, treatment efficacy is significantly reduced [3]. Consequently, there is an urgent need to investigate promising prognostic and diagnostic biomarkers.

The Cancer Genome Atlas (TCGA) has generated comprehensive panoramic maps of BC [4]. While more than 90% of the human genome is transcribed, only 1% to 2% of transcripts encode proteins. Most RNA transcripts have limited or no protein-coding capabilities. These RNAs are called non-coding RNAs (ncRNAs) [5]. Long non-coding RNAs (lncRNAs) are a category of non-coding RNAs that are longer than 200 nucleotides [6]. Increasing evidence indicates that lncRNAs play regulatory roles in various human cancers, including BC [7], breast cancer [8], squamous cell carcinoma [9], colorectal cancer [10], and others. LncRNAs have been shown to be involved in the biological processes of BC, for instance downregulation of SNHG16 causes G1 phase cell cycle arrest and promotes BC cell apoptosis [11]. LncRNA RP11-89 promotes BC cell proliferation, migration, and tumorigenesis and inhibits cell cycle arrest [12]. Exosome-transmitted lncRNA PTENP1 suppresses BC progression by increasing apoptosis and reducing invasion and migration [13]. Upregulation of MEG3 not only inhibits cell invasion and migration but also increases the chemosensitivity of BC cells to cisplatin [14].

SNHG18 (SNHG18; GenBank Accession no. NR_045196) [15] is a newly identified lncRNA located on human chromosome 5. Previous studies have reported that SNHG18 functions as a tumor suppressor in liver cancer [16], but as an oncogene in non-small cell lung cancer [17], glioma [18], and multiple myeloma [19]. However, the potential functions and mechanisms of SNHG18 in BC remains unclear. Therefore, we investigated the role of SNHG18 in the progression of BC and explored the underlying mechanisms. We found that SNHG18 is significantly downregulated in BC tissues and cell lines and is associated with patient prognosis. Through in vivo and in vitro experiments, we found for the first time that SNHG18 significantly inhibits the proliferation of BC cells. By further studying the molecular mechanism of its effect on BC cell proliferation, we found that SNHG18 delayed cell cycle progression through the SNHG18/c-Myc/p21 pathway. This study provides new insights for the treatment of BC.

## Meterials and methods

### Plasmids,antibodys,and reagents

SNHG18 was introduced into pcDNA3.1( +) using primers forword (5'-CCA CTA GTC CAG TGT GGT GGC TCC TCC TCC TCC TCC TCC-3’) and reverse (5'-CGG CCG CCA CTG TGC TGG ATT TTC AGA TTT CTA GAA TCC TTT-3’). The constructs of short hairpin RNA specifically targeting p21 (shp21) and nonsense control construct were purchased from OpenBiosystem (Thermo Fisher Scientific, NY, USA). The human p21 promoter (− 1566 to + 142) was cloned into the pGL3 basic luciferase reporter and was previously described [20]. c-Myc expression construct was kindly provided by Dr.Rosalie Sears (Oregon Health & Science University, Portland, OR, USA). Plasmids were prepared by the Plasmid Preparation/Extraction Maxi kit from QIAGEN (Valencia, CA, USA).

The antibodies specific against CyclinE2 (#4132), p-STAT1(Y701) (#7649), STAT1 (#14994), p-STAT3(Y705) (#9145), STAT3 (#30835), p-STAT5(Y694) (#4322), and STAT5 (#94205) were purchased from Cell Signaling Technology (Danvers, Massachusetts, USA). Antibodies against CDK2 (sc-6348), CDK4 (sc-60), CDK6 (sc-177), CyclinD1 (sc-20044), p21 (sc-397), p27 (sc-1641), p–c-Jun(S63) (sc-7980), p–c-Jun(S73) (sc-822), and c-Jun (sc-44) were bought from Santa Cruz Biotechnology (Santa Cruz, CA, USA). The antibody specific against β-actin (Ab0011) was bought from Abways (Shanghai, CHN). Cycloheximide (CHX) and MG132 were purchased from Calbiochem (San Diego, CA, USA).

### Cell lines and cell culture

The human BC cell lines UMUC3 and J82 were gifts from Dr. Xue-Ru Wu (Depart ments of Urology and Pathology, New York University School of Medicine). These cell lines were maintained in DMEM medium and MEM separately supplemented with 10% heat-inactivated fetal bovine serum (FBS), 1% L-glutamine, and 1% gentamycin. The human normal bladder urothelial cell line SV-HUC-1 was used in our previous publication and maintained in F12K supplemented with 10% heat-inactivated fetal bovine serum (FBS), 1% L-glutamine, and 1% gentamycin. These cells were maintained at 37 ℃ in a 5% CO_2_ incubator.

### Cell transfection and luciferase assay

Cell transfections were performed with PolyJet^™^ DNA in Vitro Transfection Reagent (SignaGen Laboratories, Rockville, MD, USA) according to the manufacturer’s instructions. Surviving cells from the antibiotics selection were pooled as stable mass transfectants. For the determination of p21 promoter-driven luciferase activity, UMUC3(Vector), UMUC3(SNHG18), J82(Vector), and J82(SNHG18) cells were each transiently co-transfected with pRL-TK together with the related promoter driven luciferase reporter. After transfection 24 h, luciferase activity was determined using a luciferase assay system kit (Promega). The results were normalized by internal TK signal. All experiments were done in triplicate, and the results are expressed as mean ± SE.

### RT-PCR

Total RNA was extracted with TRIzol reagent (Invitrogen) as described in the manufacturer’s instructions. Total RNA (5.0 μg) was used for first-strand cDNA synthesis with oligo (dT) 20 primer by Super-Script First-Strand Synthesis system (Invitrogen). Specific primers were used for PCR amplification. The primers used in this study were as follows: human SNHG18(foword, 5ʹ-CCA TCT CAG ACC AGA GGA ACA-3ʹ; reverse,5ʹ-GTG AGC AAT AAA GCA GCC CTA-3ʹ), human p21(foword, 5ʹ-GGC AGA CCA GCA TGA CAG AT-3ʹ; reverse, 5ʹ-GAT GTA GAG CGG GCC TTT GA-3ʹ), human c-Myc(foword, 5ʹ-TAC AAC ACC CGA GCA AGG AC-3ʹ; reverse, 5ʹ-AGC TAA CGT TGA GGG GCA TC-3ʹ), and human GAPDH(foword, 5ʹ-ATC AAT GGA AAT CCC ATC ACC A-3ʹ; reverse, 5ʹ-GAC TCC ACG ACG TAC TCA GCG-3ʹ).

### Western blot analysis

UMUC3 cells and J82 cells and their transfectants were seeded in 6-well plates and cultured in normal medium until 70%–80% confluence. Following culture of cells in 0.1% FBS medium for 12 h, the medium was replaced with 10% FBS DMEM or 10% FBS MEM for another 12 h. Whole-cell extracts were prepared with cell lysis buffer (10 mM Tris–HCl (pH 7.4), 1% SDS, and 1 mM Na_3_VO_4_) as described in our previous studies. Cell extracts were then subjected to western blot analysis as described previously. Images were acquired by scanning with the phosphorimager (Typhoon FLA 7000, GE Healthcare).

### Cell proliferation analysis

Cell viability was determined by utilizing the CellTiter-Glo Luminescent Cell Viability Assay Kit (Promega) according to the manufacturer’s instructions. Briefly, cells were plated in 96-well plates at a density of 1,000 cells/well and allowed to adhere overnight. The cell culture medium was then replaced with 0.1% FBS DMEM or 0.1% FBS MEM and cultured for 12 h, and the medium was then replaced with normal medium and cultured for another 1, 3, or 5 days, and then 25 μL PBS and 25μL CellTiter-Glo assay reagent was added to each well. The contents were mixed on an orbital shaker for 2 min to induce cell lysis and then incubated at room temperature for 10 min to stabilize the luminescent signal. Results were read on a microplate luminometer (LB 96 V, Berthold). Cell viability (percent) was defined as the relative absorbance of treated samples versus that of the untreated control. All experiments were performed in 96-well plates for each experiment and repeated at least three times.

### Cell invasion assay

Cell invasion function was tested using 24-well plate and cell culture insert (Corning, USA), with or without 30 μl Matrigel (BD Biosciences, USA). The upper chamber cell suspension in 200 μl medium with 0.1% FBS, the lower chamber 500ul medium with 10% FBS. After culture 24 h, the chambers were fixed with 4% paraformaldehyde and stained with Giemsa. Removed untranswelled cells from the upper chamber. For each chamber, 5 fields were randomly captured with a microscope (DMi1, Germany) and then the cell numbers were counted with Image J.

### Cell cycle analysis

The indicated cells (2 × 10^5^) were cultured in each well of 6-well plates to 70%–80% confluence with normal culture medium. Following serum starvation for 12 h, the medium was replaced with 10% FBS DMEM or 10% FBS MEM for another 12 h. Then the cells were harvested and fixed with 1 mL of ice cold 70% ethanol overnight. The fixed cells were then centrifuged (2,000 rpm, 5 min), suspended in 500μL RNase A: Propidium Iodide (1:9), and incubated for 30-60 min at room temperature. The DNA content was determined by flow cytometry using Epics XL FACS (Beckman Coulter) and CytExpert software.

### Anchorage-independent growth assay

Anchorage-independent growth in soft agar (soft agar assay) was performed as described in our earlier studies. Briefly, 1 × 10^4^ cells in 10% FBS basal medium Eagle (BME) containing 0.33% soft agar were seeded over the basal layer containing 0.5% agar in 10% FBS BME in each well of 6-well plates. The plates were incubated in a 5% CO_2_ incubator at 37 ℃ for 1–3 weeks. Colonies were captured under the DMi1 microscope (Leica Microsystems,Buffalo Grove,IL,USA), and only colonies with over 32 cells were counted. The results are presented as mean ± SD obtained from three independent experiments.

### Protein degradation experiment

UMUC3 (vector) and UMUC3 (SNHG18) cells were seeded in 6-well plates containing complete medium. When the cell density reached 80%, the medium was replaced with fresh medium containing 0.1% FBS, and cells were starved for 12 h. Then, the medium was replaced with fresh medium containing 10 μM MG-132 and 10% FBS for 5 h. After replacing the medium with fresh medium containing 50 μg/mL CHX, the plates were placed in a 37 °C incubator with 5% CO_2_. Cells were collected at 0, 1, 2, and 3 h. The c-Myc degradation rate was assessed by western blotting.

### Human BC tissue specimens

48 pairs of bladder cancer tissues and their paired adjacent normal bladder tissues were obtained from patients who underwent radical cystectomy and who were diagnosed by pathological examination. All specimens were immediately snap-frozen in liquid nitrogen after surgical removal. All patients enrolled in this study were informed and consent was given. This study was approved by the Ethics Committee of the Affiliated Hospital of WenZhou Medical University.

### Xenograft assay in nude mice

The tumor xenograft studies were performed in the Animal Institute of Wenzhou Medical University according to the protocols approved by the Medical Experimental Animal Care Commission of Wenzhou Medical University. Ten female athymic nude mice (3–4 weeks old) were purchased from Jiangsu Jicui Yaokang Biotechnology Co., Ltd. At an age of 5–6 weeks, the mice were randomly divided into different groups as indicated and then subcutaneously injected with various UMUC3 transfectants (2 × 10^6^ suspended in 100 mL PBS) in the axillary region. The nude mice were maintained under sterile conditions according to the protocol of the American Association for the Accreditation of Laboratory Animal Care. These mice were evaluated twice a week for the appearance and size of tumors, and tumors were measured with calipers to estimate the volume. Tumor sizes were evaluated using the following formula: volume (mm^3^) = (width^2^ [mm^2^] × length[mm])/2. Four weeks after cell injection, the mice were sacrificed, and the tumors were surgically removed, photographed, weighed, and used for further pathological and histopathological evaluation. None of the mice died or were sacrificed before the end of the in vivo experiment.

### Statistical analysis

Statistical analysis was performed using Prism 7.0 software (GraphPad). All data are presented as the means of triplicate assays ± SD. Student’s t test was employed to determine the significance of differences between various groups. The differences were considered significant at *p* < 0.05.

## Results

### SNHG18 is downregulated in BC and is positively correlated with survival

**Fig. 1:**
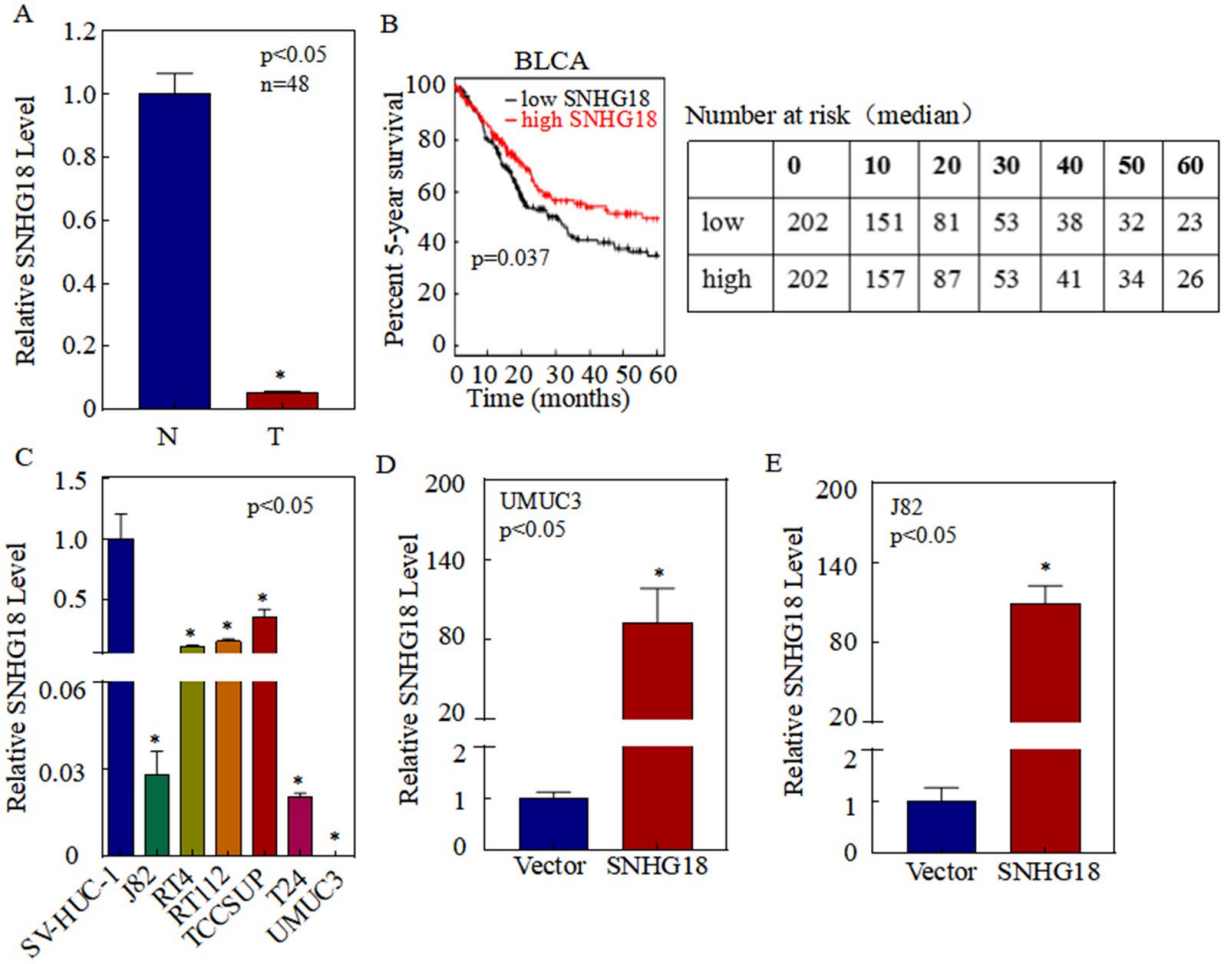
SNHG18 is downregulated in bladder cancer (BC) tissues and cell lines. **A** Relative SNHG18 expression was detected in 48 pairs of BC clinical samples by qPCR. **B** Analysis of 5-year survival rates of BC patients (*n* = 404) in The Cancer Genome Atlas database according to SNHG18 levels. **C** Relative SNHG18 expression was detected in the human normal urothelial cell line SV-HUC-1 and in the human BC cell lines J82, RT4, RT112, TCCSUP, T24, and UMUC3 by qPCR. **D**, **E** BC cell lines stably overexpressing SNHG18 were constructed. Data are presented as the mean ± SD, *p < 0.05

To uncover the role of SNHG18 in the development of BC, we examined the expression of SNHG18 in BC tissues. To this end, we collected 48 tumor and paired normal tissues, and quantitative real-time PCR (qPCR) was used to detect the relative expression of SNHG18. Compared with normal bladder tissues, SNHG18 was significantly downregulated in BC tissues (Fig. 1A).

To further investigate associations between SNHG18 expression and the prognosis of BC patients, we analyzed the relationship between SNHG18 expression and the survival of BC patients in a large public clinical microarray database using the Kaplan–Meier plotter (http://kmplot.com/). Briefly, BC patients were split into two equal groups on the basis of median SNHG18 expression and were then compared using Kaplan–Meier survival analysis. The results showed that patients with high SNHG18 expression had significantly better survival outcomes compared with patients with low SNHG18 expression (p = 0.037) (Fig. 1B).

### Ectopic SNHG18 expression inhibits BC cell proliferation in vivo and in vitro

To determine which cell lines were suitable for SNHG18 research, relative SNHG18 expression levels were analyzed in the BC cell lines J82, RT4, RT112, TCCSUP, T24, and UMUC3 by qPCR. The results showed that SNHG18 had significantly reduced expression in J82 and UMUC3 cells compared with in the human normal urothelial cell line SV-HUC-1 (Fig. 1C). Considering the low expression of SNHG18 in BC, we chose UMUC3 and J82 cells with low expression for subsequent research. To ensure the biological role of SNHG18 in BC progression, we overexpressed SNHG18 in BC cells, which was verified by qPCR. As shown in Fig. 1D (UMUC3) and 1E (J82), SNHG18-overexpressing cells (SNHG18-oe) were successfully constructed.

**Fig. 2:**
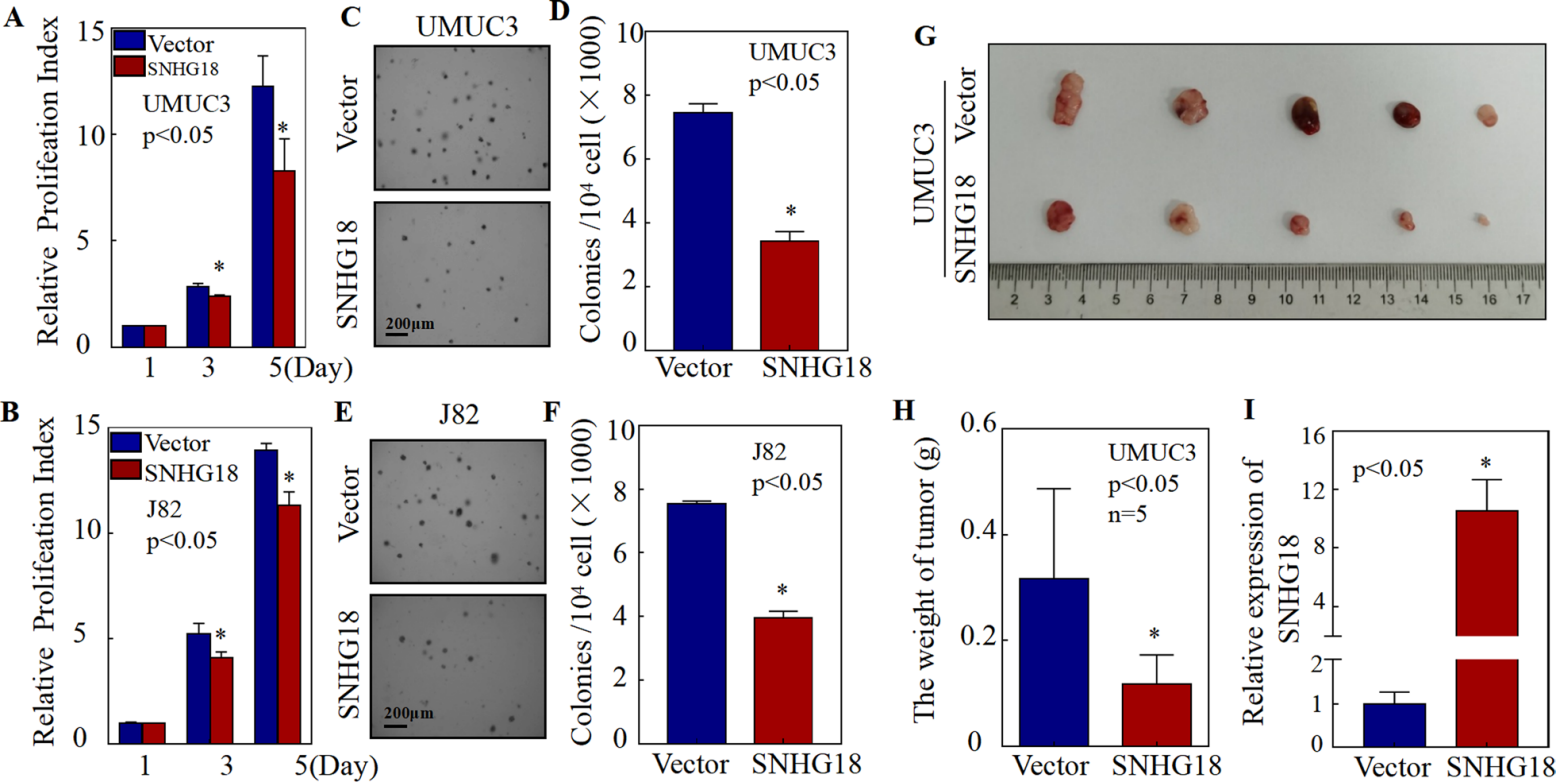
SNHG18 significantly inhibited the proliferation of bladder cancer cells in vivo and in vitro. **A**, **B** Effect of SNHG18 on the proliferation of UMUC3 **A** and J82 **B** cells, as detected by ATP assays. **C**, **D** Effect of SNHG18 on anchorage-independent growth in UMUC3 cells, as detected by soft agar assays. **C** Representative microscope images and **D** number of colonies per 10^4^ cells. **E**, **F** Effect of SNHG18 on anchorage-independent growth in J82 cells, as detected by soft agar assays. **E** Representative microscope images and **F** number of colonies per 10^4^ cells. **G**, **H** UMUC3 SNHG18-overexpressing and vector control cells were subcutaneously injected into the right back of nude mice. After 4 weeks, the mice were euthanized, and tumors were surgically removed, weighed, and photographed. **I** Total RNA was extracted from mouse xenograft tumor tissue, and qPCR was used to detect relative SNHG18 expression in tumor tissues from nude mice. Data are presented as the mean ± SD, *p < 0.05

To study the effect of SNHG18 on the proliferation of BC cells, we introduced ATP experiments and soft agar assays. The results showed that overexpressing SNHG18 dramatically reduced the monolayer proliferation ability of UMUC3 and J82 cells (Fig. 2A, B). Meanwhile, soft agar assays revealed that SNHG18 inhibited anchorage-independent growth in UMUC3 and J82 cells (Fig. 2C–F).

Considering the limitations of in vitro experiments*,* we also used the nude mouse model of subcutaneous tumor transplantation to examine the role of SNHG18 in tumor growth in vivo. UMUC3 SNHG18-oe and paired control vector cells were subcutaneously inoculated into nude mice. Approximately 4 weeks later, the mice were euthanized and tumors were harvested. Compared with the vector group, tumor volumes in the SNHG18-oe group were significantly smaller (Fig. 2G). Additionally, tumor weights were significantly reduced in the SNHG18-oe group compared with the vector group (Fig. 2H). SNHG18 expression in xenograft tumors was detected by qPCR (Fig. 2I). In addition to its function in proliferation, we also examined the role of SNHG18 in the invasive ability of BC cells. The results showed that SNHG18 expression did not affect the invasive ability of BC cells (Additional file 1: Fig. S1). Together, these in vivo and in vitro results indicate that SNHG18 inhibits the growth of BC cells.

### SNHG18 inhibits the proliferation of BC cells by upregulating p21

**Fig. 3:**
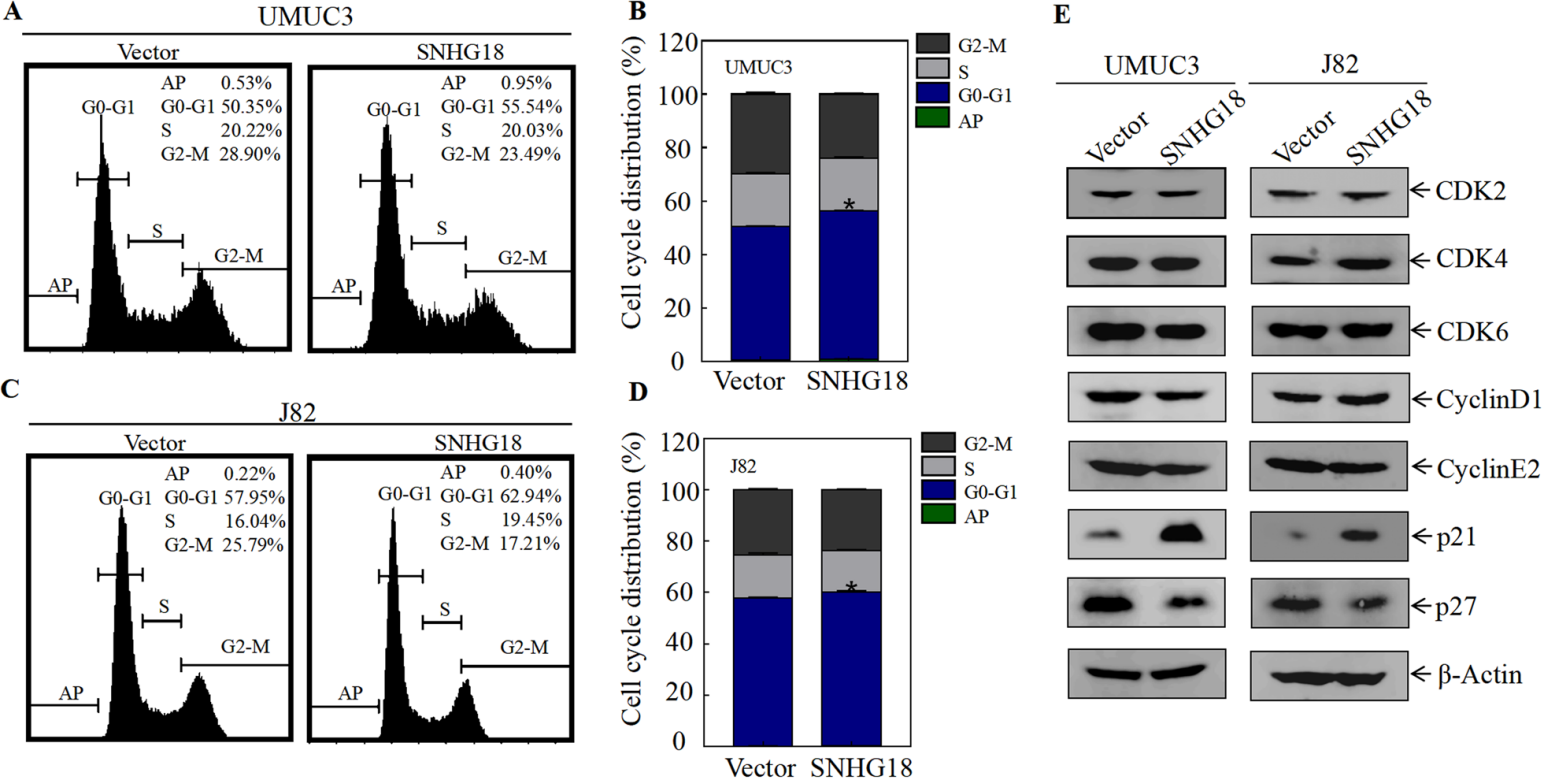
p21 acts as a downstream effector of SNHG18. **A**, **B** Flow cytometry to detect cell cycle progression of UMUC3 (vector, SNHG18). **C**, **D** Flow cytometry to detect cell cycle progression of J82 (vector, SNHG18). **E** Expression of CDK2, CDK4, CDK6, cyclin D1, cyclin E2, p21, and p27 in UMUC3 and J82 cells, as detected by western blot; β-actin was used as the internal loading control

To explore the underlying molecular mechanism through which SNHG18 inhibits BC cell proliferation, we first examined the effect of SNHG18 on cell cycle progression by flow cytometry. The results showed that compared with vector control cells, a greater percentage of SNHG18-oe cells were in G0-G1 phase, suggesting that SNHG18 inhibits the proliferation of BC cells by blocking S phase entry (Fig. 3A–D). Next, western blot analysis was performed to detect the expression of G0/G1 phase-related proteins such as CDK2, CDK4, CDK6, cyclin D1, cyclin E2, p21, and p27 [21]. As shown in Fig. 3E, SNHG18-oe UMUC3 and J82 cells consistently showed significantly upregulated p21 and downregulated p27. p21 and p27 are cyclin-dependent kinase inhibitors that negatively regulate cell cycle progression [22–25]. Taken together, p21 may participate in SNHG18-mediated inhibition of BC cell proliferation.

**Fig. 4:**
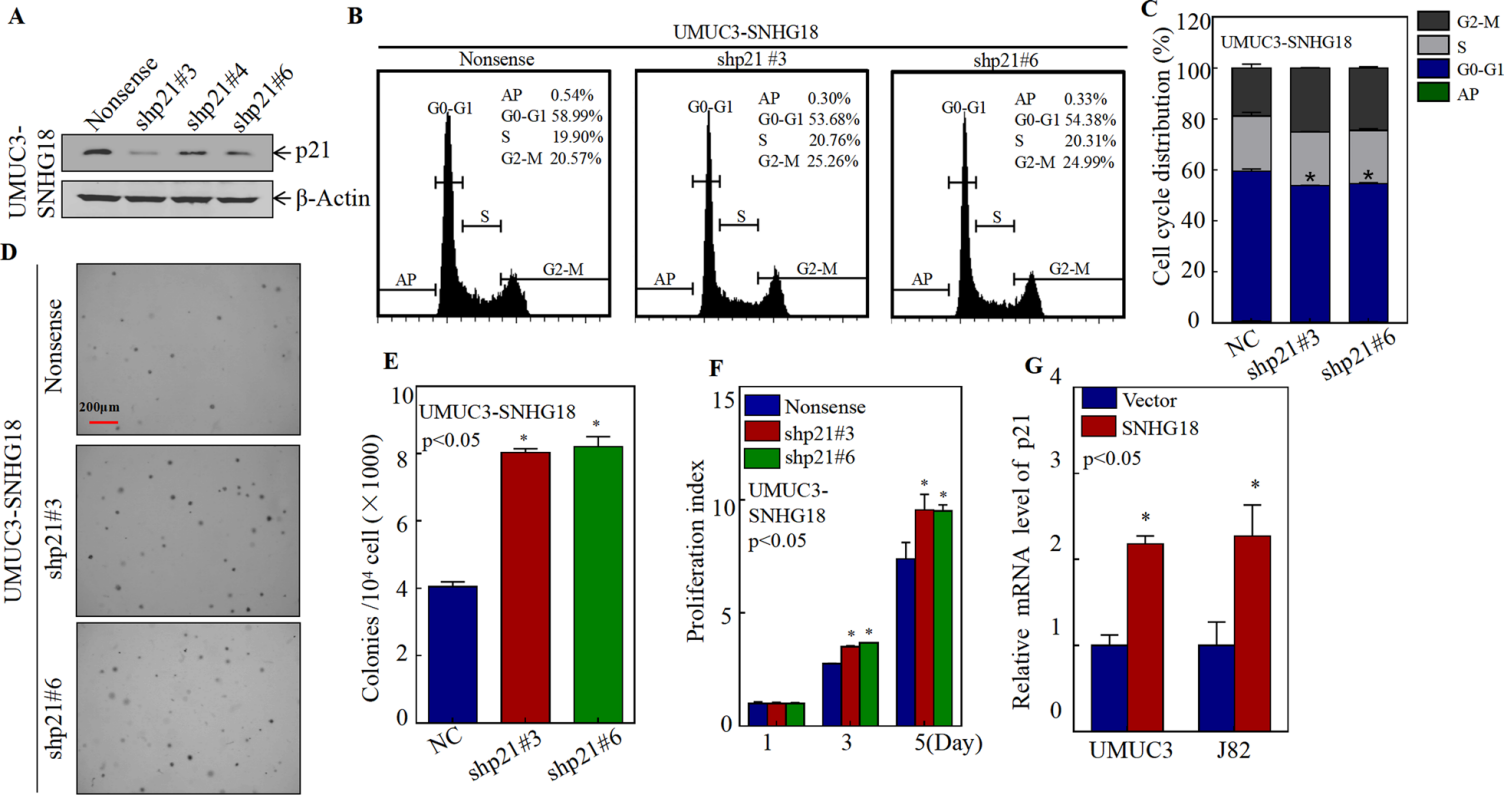
p21 inhibits the growth of BC cells, and SNHG18 promotes p21 transcription. **A** Sh-p21#3, sh-p21#4, sh-p21#6, and the control plasmid pGipz were transfected into UMUC3 SNHG18-overexpressing cells, and the knockdown efficiency was evaluated by western blotting. **B**, **C** Flow cytometry to detect cell cycle progression in UMUC3 SNHG18-overexpressing cells after knocking down p21. **D**, **E** Soft agar assays detected anchorage-independent growth in UMUC3 SNHG18-overexpressing cells after knocking down p21. **D** Representative microscope images and **E** number of colonies per 10^4^ cells. **F** ATP assays to detect the proliferation of UMUC3-SNHG18 after knocking down p21. **G** qPCR was used to detect the expression of p21 mRNA in UMUC3 (vector, SNHG18) and J82 (vector, SNHG18) cells

To assess whether SNHG18 inhibits the growth of BC cells through p21, three p21-specific shRNAs were stably transfected into UMUC3 SNHG18-oe cells (Fig. 4A). Next, cell cycle progression was detected by flow cytometry. As shown in Fig. 4B, C, the result indicated that silencing p21 abolished the G0-G1 phase cell cycle arrest. Consistently, soft agar assays showed that anchorage-independent growth was restored to SNHG18-oe cells when p21 was knocked down (Fig. 4D, E). Knocking down p21 also rescued the monolayer proliferation ability of UMUC3 SNHG18-oe cells (Fig. 4F). Together, these data suggest that p21 is the downstream effector of SNHG18.

### SNHG18 promots p21 transcription by attenuating c-Myc protein expression

**Fig. 5:**
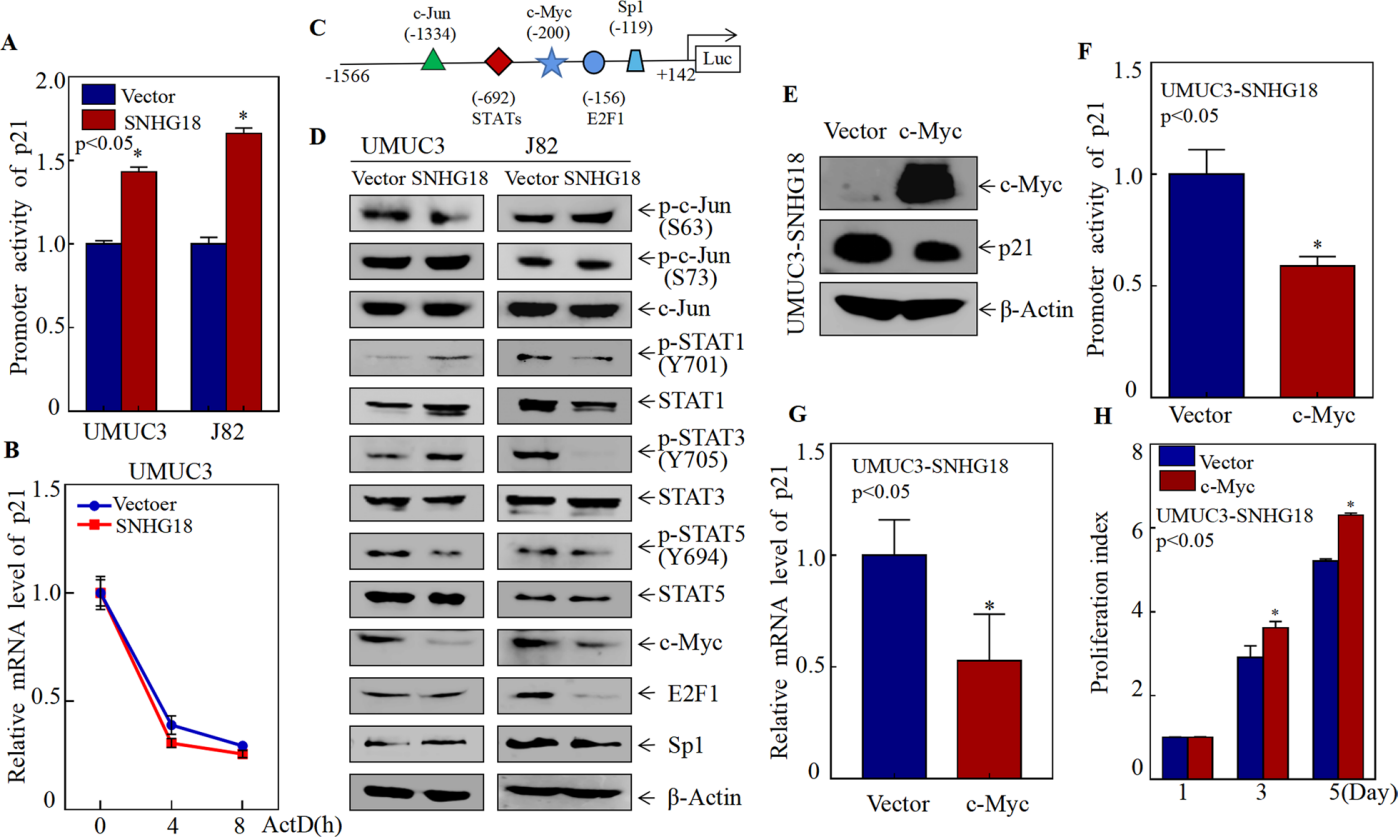
SNHG18 promoted p21 transcription by attenuating c-Myc protein expression. **A** Dual luciferase reporter assays detected p21 promoter activity in UMUC3 (vector, SNHG18) and J82 (vector, SNHG18) cells. **B** The mRNA stability of p21 in UMUC3 (vector, SNHG18) was detected. **C** Potential transcription factor binding sites in the p21 promoter. **D** Expression levels of potential transcription factors were determined by western blot. **E** The c-Myc overexpression plasmid was stably transfected into UMUC3 SNHG18-overexpressing cells, and western blotting was used to detect c-Myc and p21 protein levels. **F** Dual luciferase reporter experiments after c-Myc overexpression were used to detect changes in p21 promoter activity in UMUC3 SNHG18-overexpressing cells. **G** qPCR to detect p21 mRNA levels in UMUC3 SNHG18-overexpressing cells after restoring c-Myc. **H** ATP assays were used to detect the proliferation of UMUC3 SNHG18-overexpressing cells after rescuing c-Myc expression

To explore the mechanism through which lncRNA SNHG18 regulates p21 expression, we first examined p21 mRNA levels via qPCR. As shown in Fig. 4G, p21 mRNA levels were significantly increased in UMUC3 and J82 cells after overexpressing SNHG18, indicating that SNHG18 regulates p21 expression at the mRNA level. To determine whether SNHG18 regulates p21 expression at the level of transcription activity or mRNA stability, a p21 promoter luciferase reporter was introduced into UMUC3 and J82 cells, with TK as an internal control. The results showed that overexpressing SNHG18 increased p21 promoter-driven reporter transcriptional activity in UMUC3 and J82 cells (Fig. 5A), revealing that SNHG18 upregulates p21 by increasing transcription from its promoter. In addition, we found that overexpression of SNHG18 had no effect on the mRNA stability of p21 (Fig. 5B). Therefore, we exclude the regulation of mRNA stability.

To explore the pathway through which SNHG18 promotes p21 transcription, we used bioinformatics analysis combined with the literature [26–28]. Through this we found that potential transcription factors that could bind to the − 1566 to + 142 region of the p21 promoter included c-Jun, STATs, c-Myc, E2F1, and Sp1 (Fig. 5C). Then western blotting was applied to detect the expression of these transcription factors in both UMUC3 and J82 cells. The results showed that only c-Myc and E2F1 were inhibited by SNHG18, whereas the others showed no consistent differences (Fig. 5D). According to a previous report [29], E2F1 positively regulates transcription from the p21 promoter, which is inconsistent with the trend of increased p21 expression. Conversely, it has been widely confirmed that c-Myc is a negative regulator of p21 [30–32]. In accordance with the literature, we consider c-Myc to be more likely to participate in regulating p21 transcription downstream of SNHG18.

### SNHG18 inhibits BC cell proliferation by accelerating c-Myc protein degradation

To determine whether c-Myc was the downstream mediator responsible for SNHG18-mediated inhibition of human BC tumorigenic growth, a c-Myc overexpression plasmid was stably transfected into UMUC3 SNHG18-oe cells to rescue c-Myc expression. As shown in Fig. 5E, c-Myc overexpression was successfully achieved in UMUC3 SNHG18-oe cells. Moreover, c-Myc overexpression significantly inhibited the promoter activity and mRNA level of p21 (Fig. 5F, G). Additionally, overexpressing c-Myc restored the monolayer proliferation ability of UMUC3 SNHG18-oe cells (Fig. 5H). Collectively, these results strongly demonstrate that c-Myc is the critical transcription factor that mediates SNHG18 inhibition of BC growth by directly binding to the p21 promoter to reduce p21 expression.

**Fig. 6:**
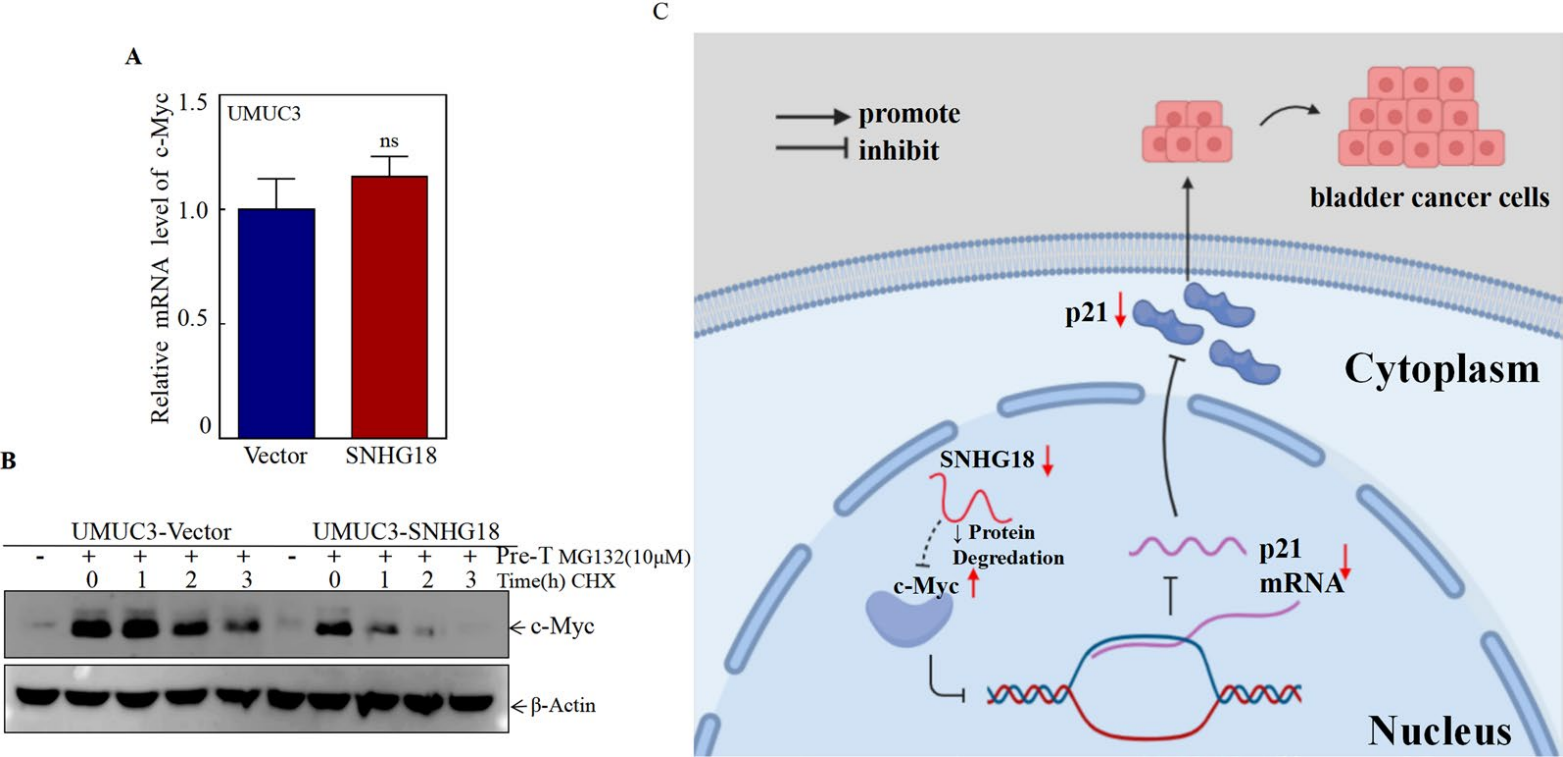
SNHG18 accelerated degradation of c-Myc protein. **A** qPCR was used to detect c-Myc mRNA levels in UMUC3 (vector, SNHG18) cells. **B** UMUC3 (vector, SNHG18) cells were pretreated with the proteasome inhibitor MG-132 (10 μM) for 8 h, after removal of MG-132, medium with CHX (50 μg/mL) was used to treat the cells for the indicated times. c-Myc protein degradation was detected by western blotting. **C** Schematic diagram of the molecular mechanism through which SNHG18 regulates the proliferation of bladder cancer cells

To investigate the mechanisms underlying SNHG18 inhibition of c-Myc protein expression, we first detected the effect of SNHG18 on c-Myc mRNA levels by qPCR. There was no significant difference in c-Myc mRNA levels between SNHG18-oe cells and vector control cells (Fig. 6A). Thus, we tested whether SNHG18 regulated c-Myc at the protein level. Protein degradation experiments were applied to test the protein half-life of c-Myc. As shown in Fig. 6B, when cells were pretreated with the proteasome inhibitor MG-132, it was found that MG-132 caused a large c-Myc accumulation. Next, MG-132 was removed, and the cells were treated with CHX at different times to test the degradation rate of c-Myc. Conversely, the degradation rate of c-Myc protein was accelerated in SNHG18-oe cells, suggesting that SNHG18 inhibits c-Myc expression by modulating the ubiquitination- proteasome pathway.

## Discussion

It has been reported that an imbalance of lncRNAs is related to the occurrence and development of BC, which indicates that lncRNAs may be a potential target for the diagnosis and treatment of BC [33–35]. SNHG18 has been reported to be associated with growth, invasion, and drug resistance in a few cancers through multiple mechanisms. SNHG18 is downregulated and can be used as an independent diagnostic index in liver cancer [16]. The high SNHG18 expression in non-small cell lung cancer is related to lymph node metastasis and a decreased overall survival rate. Mechanically, SNHG18 acts as a lncRNA mediator of MKL1 in non-small cell lung cancer, facilitating NSCLC growth and metastasis by modulating the miR-211-5p/BRD4 axis [17]. SNHG18 inhibits Semaphorin 5A (SEMA5A) through competing endogenous RNAs (ceRNAs) to promote drug resistance in gliomas, and SNHG18 expression is related to tumor degree in gliomas [15]. In patients with newly diagnosed multiple myeloma, SNHG18 and its possible target gene SEMA5A are upregulated, and high expression are related to poor prognosis [19]. These reports indicate that the specific biological effects of SNHG18 on tumor progression are tumor type-dependent, but its role in BC remains unclear.

In this study, we identified that SNHG18 is downregulated in human BC tissues and cell lines and that overexpressing SNHG18 suppressed cell proliferation. Further mechanistic studies revealed that SNHG18 may act as a tumor suppressor that exerts its antitumor effects by accelerating degradation of the transcription factor c-Myc, which allows for p21 accumulation, restraining BC development. Moreover, bioinformatics analysis showed that high SNHG18 expression was significantly correlated with good prognosis, which is indicative of the prognostic value of SNHG18 for BC patients.

The cyclin-dependent kinase inhibitor p21, also known as p21^WAF1/CIP1^, is located on chromosome 6p21.2 and encodes a 21 kDa protein. p21 inhibits the activity of all cyclin-CDK complexes with variable efficiency [36], restricting the activities of CyclinD-CDK4, CyclinD-CDK6, and CyclinE-CDK2 complexes in G0-G1 phase [24]. Although p21 plays important roles in preventing cell proliferation and promoting differentiation and senescence, recent studies have shown that p21 can promote cell proliferation and carcinogenesis under certain conditions. p21 expression depends on the cell background and environment. It can be either a tumor suppressor or an oncogene in different contexts [26]. In this study, we found that overexpressing SNHG18 upregulated the protein and mRNA levels of p21 in BC cells, inducing cell cycle arrest at G0-G1 phase. Thus, SNHG18 is an upstream regulator of p21 in BCs. Moreover, we identified that overexpressing SNHG18 resulted in increased c-Myc-dependent p21 transcription, thereby inhibiting the tumorigenic growth of BC cells, which highlights the tumor suppressor function of SNHG18 in human BC.

The proto-oncogene c-Myc is located on chromosome 8 (8q24.12-q24.13). As a strong transcription factor, it is an important regulator of cell growth, proliferation, differentiation, and apoptosis [37]. c-Myc is a negative transcription factor for p21, and ectopic c-Myc expression inhibits p21 expression by inhibiting Sp1 in the promoter region of p21. c-Myc can also inhibit p21 transcription by interfering with the activity of the transcription factor Miz-1, thus alleviating G0-G1 phase cell cycle arrest [38–40]. In addition to interfering with passive inhibition modes such as Sp1 and/or Miz-1, c-Myc can also actively recruit Dnmt3a DNA methyltransferase to the p21 promoter. These findings indicate that c-Myc can inhibit p21 transcription in a variety of ways. c-Myc can also attenuate anti-estrogen induced cell cycle arrest by inhibiting p21 [41]. Previous studies have shown that the inhibition of p21 by c-Myc may help to improve the sensitivity of tumor cells to apoptosis induced by anticancer drugs [41, 42]. Our study showed that SNHG18 inhibited the proliferation of BC cells by promoting c-Myc protein degradation and that c-Myc expression is regulated by SNHG18.

According to current studies, SNHG18 can promote c-Myc protein degradation by regulating the ubiquitin–proteasome pathway, thus inhibiting the expression of c-Myc. Increasingly, studies have proven that FBW7 mediates the degradation of c-Myc protein [43, 44]. Our next work will be devoted to exploring the molecular mechanisms through which reducing SNHG18 expression promotes c-Myc expression. Using the RNA protein interaction prediction website (http://pridb.gdcb.iastate.edu/RPISeq/), we found that SNHG18/c-Myc and SNHG18/FBW7 have the possibility of making direct RNA–protein interactions, so we speculate that SNHG18 may cooperate with the E3 ligase FBW7 to promote the degradation of c-Myc protein. However, this hypothesis needs to be further confirmed through RNA immunoprecipitation and pulldown experiments.

There are also many limitations to this study, which need to be further improved. For example, we would like to test whether SNHG18 directly interacts with c-Myc protein to accelerate its degradation, whether SNHG18 regulates the ubiquitination of c-Myc by regulating an E3 ligase, and whether SNHG18 can be used as a non-invasive liquid biopsy biomarker for BC. These need further exploration and research.

In summary, we defined the mechanism through which SNHG18 inhibits the proliferation of human BC cells by elucidating the downstream effectors of SNHG18 on the growth of BC cells. We also defined the inhibitory effect of SNHG18 on anchorage-independent growth in human BC cells in vitro and on xenograft tumors growth in vivo. Our results further indicate that overexpressing SNHG18 accelerated c-Myc degradation, which promoted p21 transcription, resulting in G0-G1 phase cell cycle arrest. Collectively, this study provides significant insight into the role of SNHG18 and suggests that SNHG18 may be a potential therapeutic target for BC.

## Conclusions

In conclusion, our study revealed low SNHG18 expression in clinical tissues and cell lines of bladder cancer, and patients with high SNHG18 expression were significantly better than patients with low expression in TCGA database, suggesting that low SNHG18 expression is an important molecular event in the development of bladder cancer. In vivo and in vitro functional experiments revealed the biological function of SNHG18 to significantly inhibit the proliferation of bladder cancer cells. Mechanism studies have shown that SNHG18 promotes the transcription and expression of p21 by inhibiting c-Myc expression, leading to G0-G1 arrest and inhibiting the proliferation of bladder cancer cells.

## Supplementary Information

**Additional file 1: Figure S1.** SNHG18 had no effect on the migration and invasion of bladder cancer cells. A, C The invasive capacity of UMUC3 (Vector, SNHG18) and J82 (Vector, SNHG18) cells were detected using Insert Membrane covered with Corning^®^Matrigel^®^. Cell migration capacity was detected using a blank Matrigel-free Insert Membrane. B, D According to the manufacturer's instructions, the invasion rate was calculated using Insert Membrane normalization. Data are presented as the mean ± SD.

## Data Availability

The data that support the findings of this study are available from the corresponding author upon reasonable request.

## Abbreviations

BC: Bladder cancer
SNHG18: LncRNA small nucleolar RNA host gene 18
TCGA: The Cancer Genome Atlas
CHX: Cycloheximide
lncRNAs: Long non-coding RNAs
ncRNAs: Non-coding RNAs
ceRNAs: Competing endogenous RNAs
