# Supplementary figures and images for: SNHG18 inhibits bladder cancer cell proliferation by increasing p21 transcription through destabilizing c-Myc protein
Source: Cancer Cell Int. 2023 Mar 16;23:48. doi: 10.1186/s12935-023-02887-w (PMC10018893; doi:10.1186/s12935-023-02887-w)

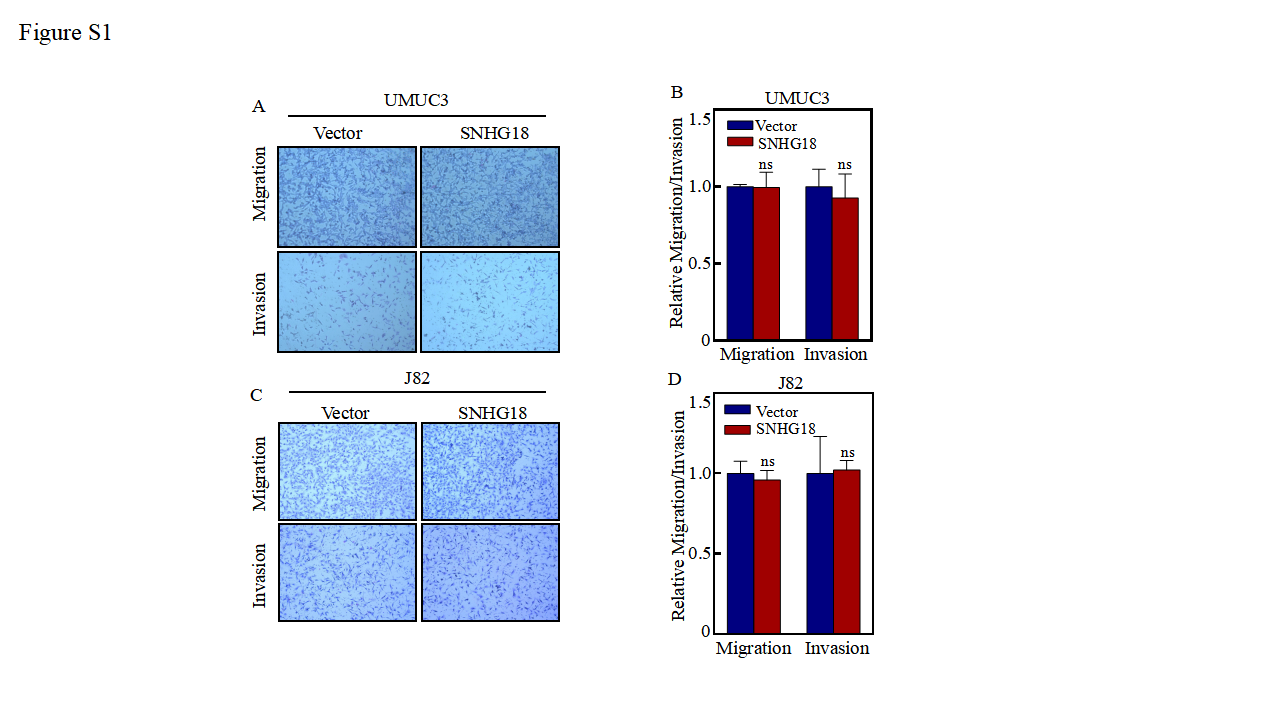

Supplement: Supplementary file 1 — Additional file 1: Figure S1. SNHG18 had no effect on the migration and invasion of bladder cancer cells. A, C The invasive capacity of UMUC3 (Vector, SNHG18) and J82 (Vector, SNHG18) cells were detected using Insert Membrane covered with Corning®Matrigel®. Cell migration capacity was detected using a blank Matrigel-free Insert Membrane. B, D According to the manufacturer's instructions, the invasion rate was calculated using Insert Membrane normalization. Data are presented as the mean ± SD. [file 12935_2023_2887_MOESM1_ESM.tif]
